# Internal Evaluation of National Leprosy Elimination Program in Tribal Gujarat

**DOI:** 10.4103/0970-0218.62574

**Published:** 2010-01

**Authors:** Anjali Singh

**Affiliations:** Department of Community Medicine, B.J. Medical College, Ahmedabad, India

**Keywords:** Evaluation, leprosy, prevalence rate

## Abstract

**Background:**

The government launched a National Leprosy Eradication program in 1983, to eliminate leprosy from India. A Modified Leprosy Elimination Campaign was started with the view to early case detection and treatment. In April 2004, a vertical program of leprosy was merged with the general health services and case detection was conducted by the general health workers in India.

**Materials and Methods:**

Internal evaluation of leprosy was done in the Panchmanal district of Gujarat through a rapid survey of the 10 Primary Health Care units in the high and low endemic areas. Active and passive surveillance data and records were verified according to the indicators.

**Results:**

Analysis of the data and record verification revealed that there was a decrease in the prevalence rate of leprosy, but it had not reached the elimination status. The MB ratio had decreased, but the child ratio remained consistent for the last five years. The disability ratio had also decreased in five years.

**Conclusion:**

The National Leprosy Elimination Program had a favorable impact, but at the same time to reach the elimination status there was a need for more stringent Information, Education, and Communication (IEC) activities to be promoted in the community. Active surveillance should be initiated so that hidden cases are not missed in the community.

## Introduction

Leprosy is one of the oldest diseases known to mankind. The word leper comes from a Greek word meaning scaly. In India, leprosy is known since ancient times as Kushta Rog, and is attributed to a punishment or curse of God. It is a chronic infectious disease caused by *Mycobacterium leprae*; an acid fast, rod-shaped bacillus. It is a highly infectious disease, but of low pathogenecity.(1) It mainly affects the skin, peripheral nerves, and occasionally the mucosa of the respiratory tract. All systems and organs can be involved in leprosy except the Central Nervous System. It is associated with grave social stigma and ostracism, which compels the patient to hide the disease and results in manifestation of deformities.

In India Multi Drug therapy (MDT) arrived in the year 1981, and was tested successfully in two pilot projects. The National Leprosy Control Program (NLCP) was then re-designated as the National Leprosy Eradication Program (NLEP), and was launched in 1983, having MDT as the core enabler.(2) On 1 April, 2004, vertical services of leprosy were integrated with the General Health Services and emphasis was given to the capacity building of the general health workers, to identify the cases and to treat them. As on December 2007, the prevalence rate in India was 0.72/10,000 population. Still India has 55% of the global case load.(3)

Gujarat achieved a status of elimination in the year 2004. At the end of August 2007, the prevalence rate in Gujarat was 0.79/10,000 population.(4) Certain districts in Gujarat have not achieved the elimination status and Panchmahal district is one of them. To achieve this remarkable achievement we needed to ensure Case detection, Quality MDT services, Treatment compliance, Awareness and Disability prevention, and so on. Under NLEP, it was decided that the internal evaluation of the program needed to be done in various districts of Gujarat. The present report is for the internal evaluation of NLEP, in the Panchmahal District, for the year 2008.

The objectives of the internal evaluation of NLEP in Panchmahal District were to identify the shortcoming in the program, recognize factors hampering the success of the program, and delineate the problems faced by various stakeholders.

## Materials and Methods

Internal evaluation was done in tribal Gujarat (Panchmahal District). A rapid survey of the PHCs of high and low endemic areas, for leprosy, was conducted, by selecting 10 PHCs randomly, out of a total 63 PHCs. The records were verified according to the indicators. The active and passive surveillance data were analyzed for the year 2007–2008 and compared with the previous years.

The indicators used for evaluation were, the prevalence rate per 10,000 population, endemicity, status of hidden cases, annual new case detection rate, proportion of the multibacillary among new cases, child ratio, disability ratio, prevalence difference ratio, status of integration, status of IEC activities, and Status of Disability Prevention and Medical Rehabilitation.

To recognize the factors for the success of the program, the annual new case detection, quality MDT services, treatment compliance, and awareness of the community, were evaluated by a rapid survey of the PHC and analyses of the active and passive surveillance data. Validation of diagnosis of a few cases of leprosy at the PHC was done, to know the accuracy of the diagnosis. A rapid survey was done in 10 PHCs to identify the hidden and untreated cases. Interviews with the general health staff and paramedics were conducted to delineate the problems faced by various stakeholders.

## Results

**Figure 1:**
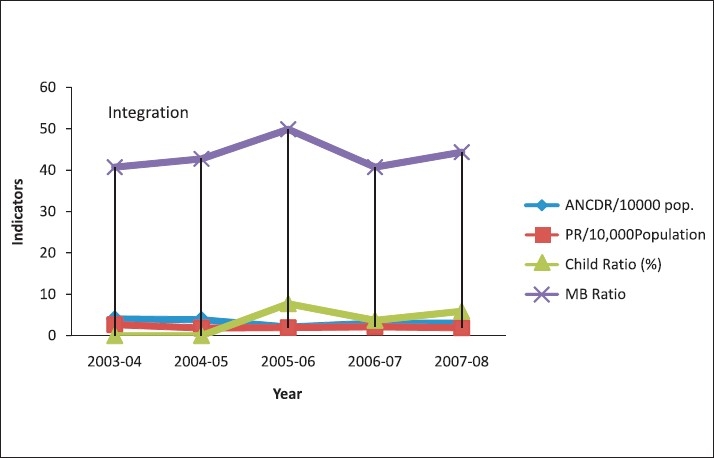
PR and ANCDR/10,000 population child ratio and MB ratio for Panchmahal district from 2003 to 2004

The prevalence rate of 2.65 per 10,000 population in the year 2003–2004 decreased to 1.87 per 10,000 population in the year 2007–2008 [Table 1, Figure 1]. There are seven talukas having a prevalence rate of more than 1 and five talukas having PR > 2 in the Panchmahal district.

**Table 1:** Performance of the district for last five years

Indicators	2003–04	2004–05	2005–06	2006–07	2007–08
New case detected	852	845	650	769	713
ANCDR	4.04	3.85	2.09	3.03	3.06
MB cases	347	361	324	313	316
MB ratio	40.72	42.72	49.84	40.70	44.32
Child cases	74	89	58	61	63
Child ratio	8.68	10.53	8.92	7.93	8.84
Deformity cases	17	44	67	15	16
Deformity ratio	1.99	5.20	10.30	1.95	2.24
Female cases	362	346	247	341	312
Female ratio	42.48	40.95	38.00	42.34	43.76
RFT	899	911	672	664	708
Pts. on treatment	560	404	364	449	443
P.R./10,000	2.65	1.84	1.92	2.13	1.90

**Figure 2:**
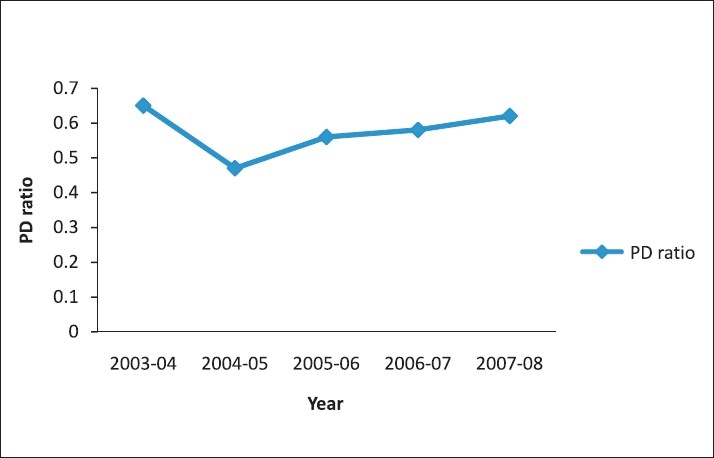
Prevalence difference ratio from 2003 to 2004, in Panchmahal District

**Figure 3:**
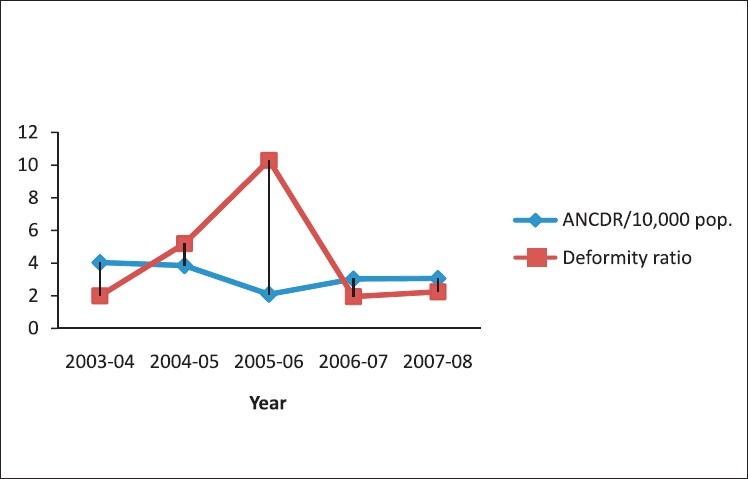
Annual new case detection rate and deformity ratio from 2003 to 2004, in Panchmahal district

The annual new case detection rate for the year 2003–2004 was 4.04, which decreased to 3.06/10,000 population, which was almost equal, but not less than the previous years. MB ratio had decreased from approximately 50% in the year 2005–2006 to 44%. In eight talukas, the MB Ratio was more than 35%. Child ratio was less than 15% in all the talukas. Child ratio had increased from 8.68% in the year 2003–2004 to 8.84% in the year 2007–2008 [Table 1, Figure 1]. The deformity ratio, which was 10.30 % in the year 2005–2006 had decreased to 2.24 % in the year 2008 [Figures 2 and 3]. The female ratio was consistent in the last five years (Approximately 43%) [Table 1].

Disability cards and LF-01 were not compiled at the sub-center level, LF-04 were not reported by the P.H.C Supervisor at some PHCs. Overall Records at the PHC such as (L.F.-01, L.F.-02, L.F.-03, L.F.-04) patient record and treatment record registers were filled properly and were quite good, however, the stock register and suspicious patient register were not filled and most of the information was lacking. In the fortnightly meeting at the PHC, held by the Medical Officer, the issue of leprosy was neither discussed nor had it been compiled in the minutes.

Community level awareness regarding leprosy is lacking. Only 20–30% of the population is aware of the disease. IEC material is not displayed at most of the PHCs. Proper IEC material is also lacking in the villages and the health workers seem least interested in the program.

There are no reconstructive surgery centers at the district; 100% of the reaction cases have been managed at the PHC. A very less percentage of people have been given a self-care kit; 15% of the patients have been provided with footwear. Out of the total 316 MB patients, one patient has developed a new deformity after MDT. No patients have been referred for surgery in the district. The vertical staff does not know the proper guidelines for ulcer care and splint's are not provided to needy patients. Involvement of the vertical staff in the Disability Prevention and Medical Rehabilitation (DPMR) project is poor; they need special training for sensitization about the project.

Drugs are properly stored and kept in proper racks, well protected from humidity and the sun; however, the drugs are not available according to the needed stock at the P.H.C and District Level.(According to the guidelines of G.O.I). Stock registers are not maintained properly at certain PHCs. Drugs are not available for patients with reaction in the store, for example, prednisolone. Expenditure for I.E.C and I.P.C activity is low. Arrangement of training and workshop for all health staff is very poor. Expenditure under different headings in the urban area is very poor.

## Discussion

The study revealed a declining trend of prevalence rate over five years, similar to a decline from 1.84/ to 0.34/10,000 population in Jamnagar between 1992 and 2001(5) and in Himachal Pradesh from 7.8 per 10,000 population in the year 1991 to 0.56 per 10,000 population in 2000.(6)

It was observed that after integration into general health services there was a decline in the Annual New Case Detection Rate (ANCDR) in the year 2005–2006, which again started increasing from the year 2006–2007. It reflects the intensification of the program activities and the efforts of training and capacity building. Similar observations were found by Mahajan *et al*. in his study.(6)

A decline in the MB ratio shows early detection of paucibacilary cases and early treatment. Surveillance activities if strengthened will again lead to a decrease in the proportion of multibacillary cases among the new cases.

Percentage of children among new cases also shows a consistent trend ranging from 8 to 11%. High prevalence of childhood leprosy shows a strong evidence of active transmission of leprosy in the community. Peat *et al*. found child ratio difficult to interpret in his study.(7)

Validation of diagnosis shows that there are few cases of multibacillary, where correct diagnosis has not been made, and they are being treated as paucibacilary cases. More focus needs to be on the decline in the prevalence difference ratio in high endemic areas in the Panchmahal district, as hidden cases are more in those areas. The deformity ratio must decrease with effective program implementation. In 2005–2006 the deformity ratio had increased, which again declined in the year 2007–2008. It also reflects that cases are detected early and timely treatment is given. It may also be due to the better awareness created with regard to leprosy, among the community. Halder *et al.* in their study found similar results.(8)

Female ratio is consistent since 2003–2004 and is around 40%, which again reflects the need for more stringent IEC activities to be promoted in the community, so that the female ratio can be brought down.

Disability prevention and medical rehabilitation needs to be strengthened. There is a need for training and sensitization of the vertical and general staff, in relation to the disability prevention and rehabilitation.

Based on the observations and analysis of indicators it is concluded that due to the implementation of the National Leprosy Elimination Program in the Panchmahal district, a favorable impact has been made on the problem of leprosy. The prevalence rate has come down from 2.65 to 1.90 /10,000 population and within a few years, the elimination of leprosy will be achieved.
